# Affective Responses by Adults with Autism Are Reduced to Social Images but Elevated to Images Related to Circumscribed Interests

**DOI:** 10.1371/journal.pone.0042457

**Published:** 2012-08-01

**Authors:** Noah J. Sasson, Gabriel S. Dichter, James W. Bodfish

**Affiliations:** 1 School of Behavioral and Brain Sciences, The University of Texas at Dallas, Richardson, Texas, United States of America; 2 Carolina Institute for Developmental Disabilities, School of Medicine, University of North Carolina at Chapel Hill, Chapel Hill, North Carolina, United States of America; George Mason University/Krasnow Institute for Advanced Study, United States of America

## Abstract

Individuals with autism spectrum disorders (ASD) demonstrate increased visual attention and elevated brain reward circuitry responses to images related to circumscribed interests (CI), suggesting that a heightened affective response to CI may underlie their disproportionate salience and reward value in ASD. To determine if individuals with ASD differ from typically developing (TD) adults in their subjective emotional experience of CI object images, non-CI object images and social images, 213 TD adults and 56 adults with ASD provided arousal ratings (sensation of being energized varying along a dimension from calm to excited) and valence ratings (emotionality varying along dimension of approach to withdrawal) for a series of 114 images derived from previous research on CI. The groups did not differ on arousal ratings for any image type, but ASD adults provided higher valence ratings than TD adults for CI-related images, and lower valence ratings for social images. Even after co-varying the effects of sex, the ASD group, but not the TD group, gave higher valence ratings to CI images than social images. These findings provide additional evidence that ASD is characterized by a preference for certain categories of non-social objects and a reduced preference for social stimuli, and support the dissemination of this image set for examining aspects of the circumscribed interest phenotype in ASD.

## Introduction

Circumscribed interests (CI) are a characteristic of autism listed within the restricted and repetitive behavior domain [1] defined by an intense preoccupation with a narrow range of subjects. CIs have been described from the earliest characterizations of autism [2] exist across all levels of symptom severity and intellectual functioning [3] [4] [5] and are ubiquitous in the disorder– an estimated 88% of individuals with an Autism Spectrum Disorder (ASD) experience CIs [6]. While this evidence suggests that CIs constitute an especially pervasive and prevalent clinical characteristic of the autism phenotype, there remains a paucity of empirical research in this area relative to other features of the disorder [7].

The content of CI can differ across individuals and may include idiosyncratic topics [6]. CI are defined more broadly by a narrow, restricted, and inflexible response set in contrast to more adaptive interests (e.g. hobbies) and by heightened motivation to pursue and remain engaged with the idiosyncratic content which can interfere with daily functioning [1] [7]. Indeed, despite the significant challenges stemming from other aspects of the disorder, parents of children with ASD cite CI as the most difficult characteristic to manage on a daily basis [8] [9], as they often require extreme patience, tolerance and accommodation [7]. Additionally, because CIs may impede the development of functional behaviors [10] [11] and peer relationships [12], and endure with age to a greater degree than other autism symptoms [9] [13] [14], they may represent a persistent and maladaptive characteristic of ASD meriting greater clinical attention and intervention [15].

The intensity and restricted focus of CI relative to non-CI stimuli suggest that abnormal cognitive-affective mechanisms may contribute to elevated rewarded value ascribed to these stimuli in ASD. Recent behavioral and neuroimaging findings support this conclusion by indicating that objects related to CI are differentially processed and prioritized by individuals with ASD. For example, adults with ASD are characterized by relative hypoactivation in neural reward circuits while anticipating monetary incentives and social stimuli but hyperactivation to images related to CI [16] [17]. These findings suggest that the presence of altered functioning of reward circuitry in ASD may constitute a potential neurobiological mechanism of CI. Consistent with this conclusion, children and adolescents with ASD exhibit restricted and preservative attention to a subset of object stimuli that are common subjects of CI (e.g., trains and electronics) [18] [19], but not to images of non-CI objects or social stimuli. Additionally, attention to social stimuli is reduced in ASD children when CI objects are concurrently displayed, but remains similar to typically developing children in the presence of non-CI objects, indicating that social attention in ASD may be modulated by the relative salience of competing stimuli [19].

Heightened saliency of CI objects in ASD may therefore result in prioritization that diminishes interest in social information, a pattern of abnormal attentional allocation that may have developmental repercussions. Although considerable research in ASD has focused on deficits in social cognition from a very early age, there has been a paucity of research directly examining the relative prioritization of social versus nonsocial information in ASD. During typical development, orientation to social stimuli begins in early infancy [20], including a neonatal preference for faces over non-face stimuli [21]. This prioritization is critical for social and language development [22] and social adaptation through the lifespan [23]. Further, attention to social stimuli, even in infancy, is hypothesized to be accompanied by feelings of pleasure and reward for typically developing individuals [24] [25], resulting in an “addiction to faces” [26] that promotes the development of neural systems underlying social information processing. In turn, such reward mechanisms may serve to encode and consolidate positive memories of social experiences [27] which may influence future responses to social stimuli. Thus, in typical development, social brain circuitry may be shaped to guide responses to social and nonsocial sources of information through a complex integrative process.

Individuals with ASD, however, may exhibit biases towards orienting and attending to nonsocial features of the environment. For example, individuals with ASD attend more to both noncritical social elements (e.g., mouths vs. eyes) [28] [29] [30] [31] (though see [32] [33] for exceptions) and nonsocial elements of social scenes (e.g., objects vs. faces) [29] [34], and demonstrate a circumscribed pattern of visual attention towards nonsocial stimuli that correlates with the magnitude of repetitive behavior symptoms [19]. Although heightened nonsocial salience is evident from a very young age in children with ASD [18] [28] [34] [35] [36] and even in infants at high risk for developing autism [37] [38] [39], it is unclear whether this abnormality originates from abnormal motivational mechanisms or a preference for low-level perceptual features such as spatial frequency and dynamic motion. However, because early emerging biases toward nonsocial information may affect the development of neural specialization [40], including neural circuitry supporting abilities related to social information processing [26] [41], abnormal prioritization of visual attention to nonsocial aspects of the environment during early development in ASD may contribute to the emergence of the phenotypic impairments of the disorder.

Thus, one potential mechanism that may explain how two seemingly disparate domains in ASD, social deficits and repetitive behaviors, co-occur in the disorder may be the presence of an abnormal cognitive-affective reward system that is “biased” away from social information towards nonsocial information. Indeed, while individuals with ASD may exhibit reduced affective responses to social information [2], including muted facial and behavioral affect accompanying social interactions [42] [43], they often exhibit displays of positive affect and enthusiasm in response to specific nonsocial aspects of the environment [44], and even engage in increased joint attention [45] and eye contact [46] when personal CI are incorporated into social interactions. This dissociation in emotional response suggests that the social and repetitive behavior domains of autism may be linked by an abnormal profile of affective experiences that is elevated in response to CI and reduced to social stimuli.

The current study sought to determine whether adults with and without ASD differed in their subjective ratings of valence and arousal on three sets of images previously developed by our research group [16] [18] [19]: (1) a set containing social content; (2) a nonsocial set containing images related to CI in autism; and (3) a nonsocial set with content unrelated to CI in autism. Valence and arousal are independent components of emotional experience that characterize affective responses to a variety of stimuli [47]. The arousal dimension reflects the extent to which an emotion is associated with a sensation of energy (i.e., calm to excited) whereas the valence dimension reflects the extent to which an emotion reflects a negative or positive state of mind subserving behavioral withdrawal or approach motivation, respectively [48] [49].

Given prior findings suggesting attention capture by “High Autism Interest” (HAI) objects related to CI in ASD [18] [19] but decreased visual attention to social stimuli [29] [34], we predicted relatively higher valence ratings of HAI images but lower valence ratings of social images by ASD relative to typically-developing (TD) adults, while the groups would not differ on valence ratings for “Low Autism Interest” LAI images (i.e., object images unrelated to CI). Such a result would indicate that HAI objects are more pleasing to individuals with ASD relative to other object types, while social stimuli are less pleasing. In contrast, we did not anticipate that these group differences would extend to arousal ratings of HAI objects, for two reasons. First, our prior work indicated when HAI images were presented in the context of an incentive delay task, individuals with ASD were characterized by relatively increased ventromedial prefrontal cortex activation during the outcome phase of the task, but not by increased nucleus accumbens activation during the anticipation phase of the task [16]. Given that incentive motivation is critically linked to the arousing properties of potential rewards, whereas the reward outcomes are linked primarily to feelings of pleasure [50] [51], these data suggest that HAI images would elicit higher valence ratings specifically. Second, this image set was derived on the basis of patterns of visual attention [18] [19]. Visual attention is mediated by a number of factors, including stimulus value [52], and animal studies have suggested that value-driven attention is mediated not by stimulus action value, but rather by stimulus valence [53], suggesting again higher valance ratings in the ASD sample. However, given that prior research has demonstrated increased physiological arousal to social stimuli in autism [54] [55], we predicted that the ASD group would provide higher arousal ratings on social stimuli.

Additionally, because the content and mechanical features of common CI may be disproportionately pleasing to males [9] [56] [57], we predicted higher valence ratings to HAI images by males than by females, regardless of group membership. However, given prior findings suggesting that HAI objects are disproportionately salient to individuals with ASD relative to social images [19], we predicted that the relative valence of HAI objects compared to social images would be greater in ASD, above and beyond any present sex effects. If found, this result would suggest that heightened valence of HAI objects relative to social stimuli may underlie previous findings of attentional prioritization of HAI objects over social images in ASD [19]. Additionally, a secondary exploratory aim was pursued to determine if a valence preference for HAI objects over SI images increased with the presence of autism-related characteristics, both within ASD and within a typically developing comparison sample. Such a finding would suggest that higher levels of autism-related traits, even at subclinical levels in unaffected typically developing populations, is associated with a disproportionate emotional preference of objects related to CI relative to social stimuli.

## Methods

### Ethics Statement

All individuals supplied written informed consent prior to study participation. The protocol for this study was approved by the UNC-Chapel Hill School of Medicine Biomedical Institutional Review Board.

### Participants

Fifty-six individuals with ASD (M age = 22.55; SD = 5.08) and 213 TD comparison individuals (M age = 21.48; SD = 3.15) participated in this study (see Table 1 for demographic information). All participants were between 18 and 39 years old and had no self-reported cognitive impairment. Recruitment of individuals with an autism spectrum condition was accomplished via email invitations to members of two North Carolina autism listserves. Thirty-nine were recruited from the University of North Carolina (UNC) Autism Research Registry and the remaining ASD participants were recruited from the Autism Society of North Carolina (ASNC). All individuals included in the UNC Autism Research Registry hold a DSM-IV clinical diagnosis of ASD, and membership on the ASNC listserve is intended for individuals self-referred as holding an autism spectrum diagnosis. Autism Quotient (AQ) [58] data were collected to confirm the presence of significant autism symptoms. ASD participants recruited from the UNC Research Registry and those recruited from the ASNC did not differ on AQ scores, nor did they differ on any outcome measure.

**Table 1:** Demographic features of participants.

	ASD group (n = 56)	TYP group (n = 213)
Variable	Mean *(SD)*	Mean *(SD)*
Age	22.55 *(5.08)*	21.49 *(3.15)*
Sex (% male)	64%	32%*
Ethnicity (% Caucasian)	84%	70%
Autism Quotient Total	31.07 *(8.98)*	16.64 *(5.74)* *

* p<.001.

TD adults were recruited via an email sent to UNC students, faculty and staff. TD participants were ineligible if they had a self-reported psychiatric diagnosis and were excluded if they had an AQ score of 32 or over, a conservative cutoff for suspected autism [58]. In total, 4 TD participants were excluded for having an AQ score of 32 or over, and 3 TD participants were excluded because of technical error.

### Procedure

This study used a set of 114 color images consisting of 40 social images, 40 High Autism Interest (HAI) images and 34 Low Autism Interest (LAI) images that have been previously described by our research group [16] [19]. All pictures were public domain photographs selected to be relatively similar in complexity and size. Social images depict a single child or adult displaying a happy expression, and both sexes and various diverse ethnicities are represented. HAI images were selected from eight categories previously reported in semi-structured parent-report interviews to be the most commonly occurring circumscribed interests in ASD: trains, electronics, vehicles, construction equipment, airplanes, clocks, blocks and road signs [6] [9]. Subsequent research has confirmed that CIs in ASD disproportionately consist of nonsocial content, especially those involving mechanical systems, vehicles, and computers [7], categories that are represented in the current image set and also relate to common interests found in typically developing males [57]. Prior studies by our group have validated that these objects are of disproportionate salience to ASD individuals, eliciting greater visual attention within a passive-viewing exploration task [18] [19]. The 40 objects that demonstrated the highest number of visual fixations from Sasson et al [19] were used in this study. LAI images consisted of eight categories of everyday objects not commonly associated with CI: clothing, outerwear, office supplies, kitchen supplies, furniture, tools, musical instruments and plants. All images were modified to a resolution of 300 pixels per inch, a width of 500 pixels, a height of 400 pixels.

### Testing Procedure

All data in this study were collected via web-based questionnaires and ratings procedures completed by participants. We chose to administer the task online because it enabled us to collect data from a large enough sample to allow for detailed analyses. These additional analyses included examining sex differences between and within groups, as well as associations between affective ratings and the presence of autism-related characteristics. Interested participants clicked on a survey link sent via email. The survey site first presented informed consent information followed by the AQ and several demographic questions (e.g., age, sex, and ethnicity). Next, participants were presented with instructions about how to make emotional ratings of the images they would see. They were instructed to provide two ratings for each image, one for valence and one for arousal, using the Self-Assessment Manikin (SAM) [59]. The SAM is a nonverbal graphical assessment measuring affective responses of valence and arousal that has been used to normalize and anchor responses in many prior studies. Two nine-point scales, one for valence and one for arousal, were presented along with a series of schematic characters depicting the range of possible responses. Participants selected the point on each scale that corresponded to their emotional response to each presented image. Participants were informed that the range of valence ratings was from −4 to +4 and reflected “how good or bad the picture makes you feel. So, if the picture makes you feel sad, rate it negative. If the picture makes you feel happy, rate it positive”. Participants were informed that the range of arousal ratings was from 0 to 8 and reflected “how calm or excited the picture makes you feel. If you find the picture makes you feel calm, you would rate it lower. If you find the picture makes you feel energetic or excited, you would rate it higher”. Participants were also told that they would rate 114 images, and that because there was no right or wrong answer, they should respond as honestly and as quickly as possible. All participants were required to indicate that they understood the task and rating scales before beginning. Images were then presented one at a time in a randomized order. Each image was presented along with both SAM ratings scales displayed sequentially (first valence, then arousal) underneath the image. The SAM rating scales were displayed along with each image in order ensure that participants remained conscientious about the meaning and scales of the valence and arousal ratings throughout the entire task. Trials were self-paced, and participants had as long as needed to make their ratings. After each arousal rating was made, the next image appeared, and this process repeated until all 114 images had been rated. It was not possible to skip items. Participants were compensated with a $20 gift card for participation.

### Statistical Analysis

Because there were no significant differences between groups for chronological age, *t* (66.45) = 1.50, p = .14 or race, χ^2^ (5, *N* = 269) = 7.91, p = .16, these variables were not considered in subsequent analyses. However, the groups differed on sex distribution, χ^2^ (1, *N* = 269) = 20.23, p<.001). Because ASD occurs over four times more often in males than in females [60], this imbalance was driven primarily by the challenges related to identifying and recruiting females with ASD, who we oversampled (n = 20; 36% of the sample) in order to examine sex differences within and between groups. We also included a large sample of TD females in order to test hypotheses related to sex differences in the general population, and entered sex as a between groups variable to determine if it predicted affective responses independent of clinical status. When Levene's test indicated unequal variances between groups during independent samples t-tests, statistics are reported with equal variances not assumed. When Mauchly's test indicated a violation of the assumption of sphericity, Greenhouse-Geisser corrections were employed.

The primary hypothesis of the current study predicted that, relative to TD participants, ASD individuals would provide higher valence ratings for HAI objects and lower valence ratings for social images, while the groups would not on differ on valence ratings for LAI objects, or on arousal ratings for any image type. Because arousal and valence are conceptually and psychometrically separable components of emotion [47], hypotheses were tested by conducting separate mixed model ANOVAs for each with group (ASD vs. TD) and sex (M vs. F) as the between group variables and image type (HAI vs. LAI vs. Social) as the within group variable. Significant between-group effects were followed up with post hoc t-tests and univariate ANOVAs.

We further predicted that, independent of sex, the ASD group would differ from the TD group in *relative* ratings, such that they would rate HAI objects as higher on valence than social images, while TD individuals would not demonstrate this discrepancy. Such a finding would provide support that ASD is characterized by a greater positive affective response to HAI objects than to social images, even after controlling for anticipated sex effects (i.e., heightened valence ratings for males relative to females). This pattern of group differences was not anticipated for the discrepancy in arousal ratings between social images and HAI. To test this hypothesis, discrepancy scores (aka difference scores) were computed by calculating the difference in ratings between social images and HAI objects for both arousal and valence, and a multivariate ANOVA with group (ASD vs. TD) as the fixed factor, sex as a covariate, and discrepancy ratings for valence and arousal as the dependent variables. Finally, to explore whether the presence of subclinical traits of autism within the TD group was positively associated with valence preferences for HAI images relative to social images, correlation analyses were conducted between AQ scores and the Social minus HAI image valence rating discrepancy score.

## Results

Although valence and arousal are conceptualized as theoretically distinct components of emotion and thus analyzed separately, inter-correlations were conducted for statistical confirmation within the current sample. Valence and arousal ratings across image categories were significantly but only minimally correlated (*r* = .16, p = .01), suggesting that these dimensions were indeed largely distinct across groups.

*Arousal Ratings:* Arousal ratings for all HAI, LAI and social images, sub-divided by group and sex, can be seen in Tables 2, 3 and 4, respectively.

**Table 2:** Means and standard deviations for arousal ratings on HAI images.

		Image #	TD Males	TD Females	ASD Males	ASD Females
*Trains*			4.48 (1.19)	4.33 (.83)	4.57 (1.13)	4.47 (1.68)
	Train 1	15	4.64 (1.69)	4.38 (1.31)	4.75 (1.15)	4.50 (2.01)
	Train 2^B, C^	31	4.95 (1.47)	4.22 (1.28)	4.83 (1.52)	4.80 (2.17)
	Toy Train	33	4.19 (1.64)	4.27 (1.22)	4.50 (1.50)	3.90 (2.77)
	Train 3	35	4.07 (1.74)	4.23 (1.04)	4.08 (1.87)	4.10 (1.97)
	Train 4	37	4.88 (1.59)	4.55 (1.36)	4.67 (1.48)	5.05 (1.50)
*Electronics*			5.36 (1.48)	5.07 (1.19)	5.32 (1.67)	5.32 (1.32)
	iPhone	10	5.78 (1.91)	5.59 (1.63)	5.33 (1.62)	5.80 (1.82)
	Nintendo 1	14	5.72 (1.53)	5.42 (1.74)	5.19 (2.16)	6.15 (1.76)
	Nintendo 2	18	4.91 (2.01)	4.72 (1.66)	5.31 (2.04)	4.70 (2.56)
	Nintendo 3	32	5.06 (1.75)	4.79 (1.45)	5.22 (2.03)	5.25 (2.49)
	X-box^A^	34	5.36 (1.94)	4.81 (1.51)	5.53 (2.05)	4.70 (2.30)
*Vehicles*			4.76 (1.02)	4.57 (.88)	4.59 (1.08)	4.46 (1.14)
	School Bus	1	4.09 (1.68)	4.19 (1.38)	4.36 (1.76)	4.15 (1.87)
	Sports Car 1	21	6.03 (1.76)	5.42 (1.32)	5.33 (1.69)	5.40 (1.90)
	Sedan	23	4.01 (1.56)	4.12 (1.51)	4.36 (1.53)	3.25 (2.00)
	SUV	30	4.67 (1.45)	4.38 (1.48)	4.31 (1.65)	4.30 (1.81)
	Sports Car 2	39	5.00 (1.53)	4.76 (1.63)	4.61 (1.48)	5.20 (2.12)
*Construction* ^A^			4.28 (1.11)	4.08 (.83)	4.34 (1.14)	3.54 (1.42)
	Bulldozer 1^A^	2	4.51 (1.79)	4.13 (1.32)	4.44 (1.36)	3.95 (2.11)
	Bulldozer 2	7	4.34 (1.73)	4.12 (1.11)	4.42 (1.42)	3.90 (1.71)
	Tractor 1^A^	25	4.34 (1.31)	4.14 (1.05)	4.36 (1.22)	3.30 (2.00)
	Forklift	28	4.24 (1.46)	4.08 (1.23)	4.36 (1.38)	3.50 (1.91)
	Tractor 2	38	3.97 (1.74)	3.90 (1.19)	4.11 (1.43)	3.05 (1.88)
*Airplanes* ^A^			5.29 (1.20)	4.76 (.98)	5.27 (1.26)	4.77 (1.44)
	Plane 1^A^	9	4.94 (1.62)	4.60 (1.37)	5.22 (1.66)	4.40 (1.79)
	Jet^A^	19	5.70 (1.86)	4.75 (1.48)	5.64 (1.64)	5.00 (1.97)
	Plane 2^A^	20	5.21 (1.53)	4.74 (1.50)	5.11 (1.72)	4.50 (2.14)
	Helicopter^A^	27	5.06 (1.70)	4.65 (1.38)	5.25 (1.54)	4.90 (1.94)
	Shuttle	29	5.54 (1.59)	5.10 (1.54)	5.11 (2.03)	5.05 (2.06)
*Blocks* ^C^			4.24 (.97)	4.37 (.92)	4.17 (1.13)	3.58 (1.71)
	Blocks 1	4	4.28 (1.77)	4.77 (1.36)	4.47 (1.70)	4.25 (2.20)
	Legos	6	4.54 (1.75)	4.45 (1.55)	4.00 (1.69)	4.00 (2.22)
	Blocks 2^C^	13	4.82 (1.72)	4.88 (1.53)	4.33 (1.97)	3.55 (2.26)
	Blocks 3	16	4.31 (1.51)	4.40 (1.29)	4.17 (1.54)	3.85 (2.16)
	Blocks 4	22	3.25 (1.69)	3.38 (1.49)	3.86 (1.27)	2.25 (1.83)
*Clocks*			4.38 (1.10)	4.54 (.77)	4.59 (1.23)	4.13 (1.17)
	Watch 1	5	4.49 (1.66)	4.82 (1.30)	4.58 (1.78)	3.95 (2.04)
	Watch 2	8	4.37 (1.94)	4.22 (1.29)	4.42 (1.44)	3.80 (1.99)
	Big Ben	11	4.33 (1.78)	4.76 (1.87)	4.36 (1.81)	4.70 (1.78)
	Alarm Clock	12	4.24 (1.78)	4.68 (1.57)	4.75 (1.75)	3.75 (1.41)
	Watch 3	17	4.48 (1.64)	4.23 (1.31)	4.86 (1.90)	4.45 (2.16)
*Signs*			4.24 (1.03)	4.29 (.85)	4.26 (.95)	3.65 (1.24)
	Interstate	3	4.45 (1.82)	4.49 (1.29)	4.28 (1.52)	3.80 (2.07)
	No U-Turn	24	4.27 (1.38)	4.27 (1.33)	4.25 (1.25)	3.80 (1.44)
	Dead End	26	4.15 (1.71)	4.26 (1.55)	4.06 (1.90)	3.55 (2.01)
	Yield	36	4.07 (1.51)	4.29 (1.16)	4.42 (1.40)	3.30 (1.72)
	One Way	40	4.24 (1.35)	4.16 (1.42)	4.31 (1.27)	3.80 (1.79)

p<.05: A: Males>Females; B: ASD>TD; C: TD>ASD.

**Table 3:** Means and standard deviations for arousal ratings on LAI images.

		Image #	TD Males	TD Females	ASD Males	ASD Females
*Clothing*			3.64 (1.08)	3.68 (.83)	4.01 (.90)	3.14 (1.40)
	Pants	2	3.37 (1.49)	3.38 (1.31)	4.00 (1.24)	3.30 (1.69)
	Shirt	12	3.76 (1.47)	3.83 (1.17)	4.17 (1.42)	3.55 (1.70)
	Jeans	17	3.87 (1.71)	3.88 (1.29)	3.92 (1.27)	3.10 (2.02)
	Shoe	33	3.54 (1.63)	3.88 (1.33)	4.08 (1.13)	3.89 (2.26)
	Socks^B^	34	3.67 (1.85)	3.42 (1.30)	3.86 (1.38)	2.45 (2.19)
*Outerwear*		4.03 (1.05)	3.94 (.79)	3.98 (.72)	3.76 (1.49)	
	Gloves	11	3.67 (1.73)	3.57 (1.29)	3.81 (1.41)	3.40 (2.41)
	Hat	15	4.07 (1.48)	3.84 (1.07)	4.14 (.79)	3.60 (1.93)
	Jacket	16	3.85 (1.62)	4.03 (1.33)	3.67 (1.67)	3.65 (2.25)
	Sunglasses	37	4.52 (1.62)	4.33 (1.33)	4.31 (1.49)	4.45 (1.43)
*Office Supplies*		3.81 (.93)	4.09 (.76)	4.11 (.94)	3.53 (.99)	
	Pencils^C^	5	4.27 (1.30)	4.73 (1.77)	4.17 (2.09)	3.00 (2.43)
	Key	18	3.72 (1.88)	3.91 (1.26)	4.14 (1.25)	3.95 (1.36)
	Lock	21	3.67 (1.41)	3.73 (1.31)	3.81 (1.45)	3.95 (1.90)
	Pens	26	3.43 (1.53)	3.87 (1.28)	4.36 (1.48)	3.45 (1.93)
	Scissors	32	3.94 (1.34)	4.21 (1.22)	4.08 (1.27)	3.30 (2.00)
*Kitchen Supplies*		3.89 (1.19)	3.86 (1.00)	3.92 (1.16)	3.45 (1.78)	
	Bowl	30	4.36 (1.94)	4.36 (1.65)	4.11 (1.37)	3.60 (2.46)
	Sponge	35	3.51 (1.71)	3.40 (1.44)	3.61 (1.46)	3.30 (1.63)
	Teapot	39	3.79 (1.60)	3.82 (1.63)	4.03 (1.54)	3.45 (1.93)
*Furniture*		3.07 (1.42)	3.33 (1.04)	3.47 (1.30)	2.69 (1.23)	
	Chair	3	2.58 (1.91)	2.95 (1.62)	3.11 (1.83)	3.11 (1.76)
	Chest	4	3.19 (1.76)	3.47 (1.37)	3.69 (1.45)	2.35 (1.76)
	Drawers	7	3.13 (1.61)	3.42 (1.50)	3.56 (1.40)	2.35 (1.98)
	Table	38	3.36 (1.63)	3.47 (1.24)	3.50 (1.65)	3.45 (1.88)
*Tools*			3.97 (.98)	4.00 (.78)	4.04 (.91)	3.54 (1.39)
	Flashlight	9	3.97 (1.30)	3.97 (1.14)	4.28 (1.52)	3.30 (1.52)
	Hammer	14	4.10 (1.58)	4.18 (1.32)	4.06 (1.33)	3.85 (2.00)
	Toolbox	22	3.99 (1.43)	3.91 (1.18)	3.92 (1.16)	3.50 (2.04)
	Brush	25	3.88 (1.50)	4.02 (1.41)	3.86 (1.22)	4.00 (2.32)
	Wrench	40	3.91 (1.58)	3.91 (1.25)	4.08 (1.11)	3.05 (1.93)
*Instruments^C^*		4.56 (1.11)	4.74 (1.02)	4.39 (1.18)	4.11 (1.67)	
	Drums	8	4.73 (1.55)	5.03 (1.49)	4.97 (1.87)	4.30 (2.41)
	Guitar	13	4.48 (1.80)	4.23 (1.76)	4.06 (1.80)	3.75 (2.47)
	Piano	19	4.06 (1.70)	4.25 (1.66)	4.00 (1.82)	4.00 (2.49)
	Maracas^A, C^	23	4.99 (1.65)	5.46 (1.43)	4.53 (1.34)	4.40 (1.81)
*Plants*			3.26 (1.24)	3.61 (1.21)	3.52 (1.20)	3.09 (1.51)
	Flowers	10	3.39 (2.04)	4.01 (1.93)	3.78 (1.53)	3.70 (2.18)
	Leaf	20	3.39 (1.90)	3.75 (2.19)	3.11 (1.69)	3.15 (2.47)
	Pine cone	27	3.46 (1.57)	3.81 (1.48)	3.72 (1.54)	2.95 (1.76)
	House Plant	29	2.79 (1.64)	2.86 (1.52)	3.47 (1.68)	2.55 (1.82)

p<.05: A: Females>Males; B: Males>Females; C: TD>ASD.

**Table 4:** Means and standard deviations for arousal ratings on Social images.

		Image #	TD Males	TD Females	ASD Males	ASD Females
*Children* ^D^			4.21 (1.16)	4.57 (1.15)	4.23 (1.04)	3.63 (1.91)
	Infant 1^D^	1	4.58 (2.10)	5.00 (2.07)	4.39 (1.79)	3.75 (2.31)
	Infant 2	2	4.13 (1.64)	4.58 (1.80)	4.44 (1.63)	3.50 (2.56)
	Infant 3^B, D^	3	4.28 (1.92)	4.91 (1.96)	4.03 (1.36)	3.80 (2.50)
	Boy 1^B^	4	3.79 (1.47)	4.42 (1.49)	4.19 (1.17)	3.55 (2.01)
	Boy 2	5	4.16 (1.51)	4.29 (1.70)	4.11 (1.12)	3.65 (2.50)
	Girl 1	8	4.15 (1.64)	4.31 (1.75)	4.14 (1.62)	3.70 (2.03)
	Girl 2	9	4.60 (1.77)	4.63 (1.79)	4.36 (1.44)	3.85 (2.11)
	Girl 3	10	4.22 (1.50)	4.57 (1.51)	4.31 (1.45)	3.75 (2.31)
	Girl 4	11	3.97 (1.59)	4.39 (1.41)	4.06 (1.39)	3.45 (1.79)
*Adult Men*			3.91 (.88)	4.10 (.66)	4.43 (1.08)	3.62 (1.51)
	Man 1	6	3.96 (1.80)	3.99 (1.71)	4.69 (1.80)	4.05 (1.82)
	Man 2	7	5.00 (1.71)	4.78 (1.30)	5.14 (.93)	4.45 (2.21)
	Man 3	12	3.63 (1.61)	3.87 (1.16)	4.28 (1.60)	3.05 (1.73)
	Man 4	13	3.96 (1.51)	3.95 (1.13)	4.31 (1.31)	3.25 (1.83)
	Man 5^C^	14	3.63 (1.24)	3.76 (1.18)	4.56 (1.05)	3.40 (1.60)
	Man 6	15	3.52 (1.70)	3.95 (1.27)	4.28 (1.26)	3.40 (1.96)
	Man 7^C^	16	3.76 (1.68)	3.79 (1.48)	4.47 (1.70)	3.95 (2.35)
	Man 8	17	3.69 (1.42)	3.75 (1.18)	3.94 (1.51)	3.20 (1.94)
	Man 9	18	4.25 (1.67)	4.62 (1.41)	4.53 (1.48)	3.75 (2.05)
	Man 10	19	3.87 (1.50)	4.27 (1.15)	4.39 (1.36)	3.60 (1.67)
	Man 11^C^	20	3.67 (1.70)	3.92 (1.24)	4.81 (1.58)	3.50 (2.24)
	Man 12	21	4.07 (1.39)	4.51 (1.31)	4.39 (1.40)	3.40 (1.50)
	Man 13	22	3.93 (1.32)	4.33 (1.30)	4.56 (1.56)	3.70 (1.78)
	Man 14	23	3.79 (1.40)	3.87 (1.04)	3.86 (1.40)	3.55 (1.76)
	Man 15	24	4.03 (1.44)	4.05 (1.23)	4.31 (1.26)	3.70 (2.05)
	Man 16	25	3.87 (1.80)	4.14 (1.43)	4.33 (1.45)	4.00 (1.89)
*Adult Women* ^A^		4.26 (1.07)	3.94 (.66)	4.32 (1.04)	3.50 (1.27)	
	Woman 1	26	4.03 (1.42)	3.87 (1.38)	3.92 (1.30)	3.00 (1.75)
	Woman 2^C^	27	3.79 (1.68)	3.78 (1.16)	4.53 (.65)	3.60 (1.73)
	Woman 3	28	4.01 (1.31)	3.82 (1.13)	4.17 (1.61)	3.35 (1.87)
	Woman 4	29	3.87 (1.56)	4.07 (1.34)	4.22 (1.33)	3.70 (2.20)
	Woman 5	30	4.06 (1.50)	3.88 (1.07)	4.22 (1.24)	3.40 (1.82)
	Woman 6^A^	31	3.79 (1.52)	3.54 (1.26)	4.19 (1.41)	3.55 (1.93)
	Woman 7^A^	32	5.49 (1.65)	4.08 (1.27)	4.61 (1.46)	4.25 (2.00)
	Woman 8^A^	33	4.22 (1.50)	3.77 (1.08)	4.14 (1.55)	3.05 (1.88)
	Woman 9	34	3.66 (1.61)	3.90 (1.17)	4.31 (1.31)	3.30 (1.72)
	Woman 10^A, D^	35	5.21 (1.88)	4.69 (1.37)	4.69 (1.58)	3.60 (1.88)
	Woman 11^A^	36	5.12 (1.92)	3.84 (1.15)	4.58 (1.42)	3.65 (2.16)
	Woman 12^A^	37	3.81 (1.23)	3.68 (1.11)	4.17 (1.23)	3.00 (1.75)
	Woman 13	38	3.96 (1.54)	4.11 (1.18)	4.33 (1.22)	3.55 (2.01)
	Woman 14^A^	39	4.91 (1.63)	4.10 (1.22)	4.53 (1.28)	3.60 (1.93)
	Woman 15	40	4.04 (1.45)	3.98 (1.14)	4.14 (1.27)	3.90 (1.21)

p<.05: A: Males>Females; B: Females>Males; C: ASD>TD; D: TD>ASD.

**Figure 1:**
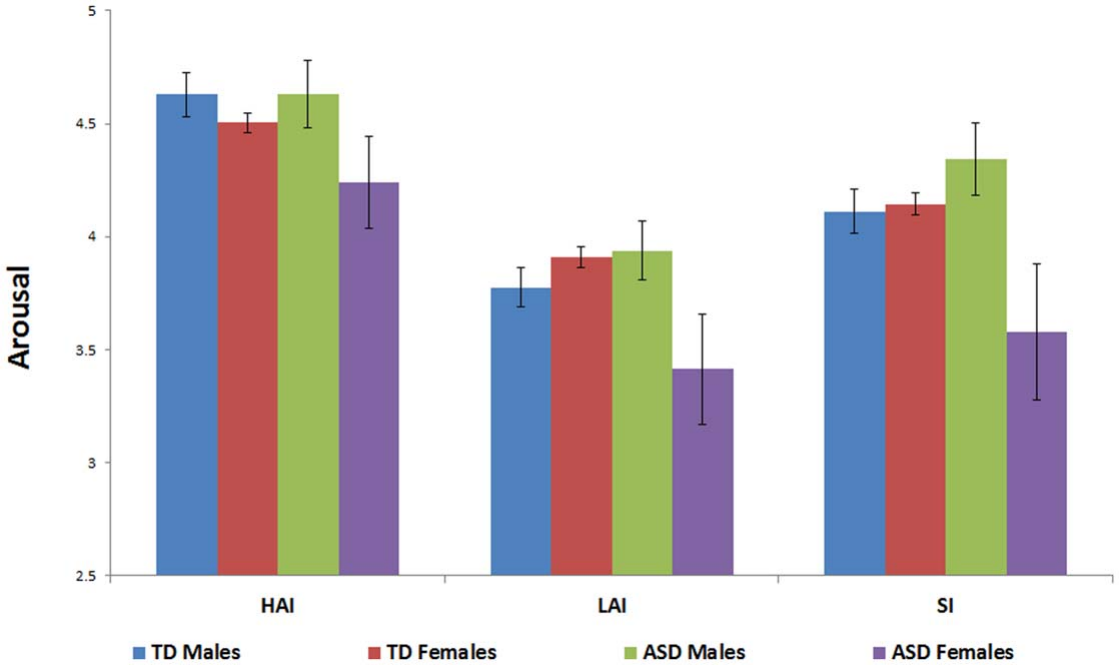
Arousal ratings for social images, HAI objects and LAI objects, by group and gender.

A main effect of image type was found (F (1.90, 504.99) = 106.97, p<.001, eta squared = .29; see Figure 1). Post hoc paired-samples t-tests revealed that HAI images were rated significantly higher on arousal than both LAI images (t (268) = 18.63, p<.001) and social images (t (268) = 9.38, p<.001), and social images were rated significantly higher than LAI objects (t (268) = 7.26, p<.001). Consistent with hypotheses, arousal ratings did not differ by group (p = .11). A main effect of sex (F (1, 254) = 5.69, p = .01, eta squared = .03) indicated that males provided overall higher arousal ratings than females, and a significant group by sex interaction (F (1, 254) = 6.23, p = .003, eta squared = .03) emerged that was driven by TD females providing significantly higher arousal ratings than ASD females (t (164) = 3.38, p = .001) and ASD males giving significantly higher arousal ratings than ASD females (t (54) = 2.31, p = .03), while all other group x sex comparisons were not significant. Neither group (p = .89) nor sex (p = .26) interacted with image type, but the three way interaction between them was significant (F (1.91, 504.99) = 3.49, p = .03, eta squared = .01), with ASD females providing disproportionately lower arousal ratings for LAI objects and social images relative to ASD males, TD males and TD females.

*Valence Ratings:* Valence ratings for all HAI, LAI and social images, sub-divided by group and sex, are presented in Tables 5, 6 and 7.

**Table 5:** Means and standard deviations for valence ratings on HAI images.

		Image #	TD Males	TD Females	ASD Males	ASD Females
*Trains^A,, B^*			.77 (1.20)	29 (.98)	.79 (1.12)	1.13 (1.17)
	Train 1^A, B^	15	.82 (1.45)	08 (1.42)	80 (1.33)	1.05 (1.43)
	Train 2^A, B^	31	.69 (2.50)	−.03 (1.23)	66 (1.51)	1.35 (1.56)
	Toy Train	33	.82 (1.54)	.76 (1.48)	1.14 (1.54)	1.10 (1.71)
	Train 3^A, B^	35	.64 (1.43)	.23 (1.26)	.80 (1.53)	.85 (1.27)
	Train 4	37	.87 (1.77)	.42 (1.42)	.57 (1.56)	1.30 (1.66)
*Electronics* ^B^			1.62 (1.48)	1.12 (1.37)	1.48 (1.70)	2.06 (1.27)
	iPhone	10	1.84 (1.90)	1.61 (1.69)	1.54 (1.84)	2.35 (1.76)
	Nintendo 1	14	1.93 (1.78)	1.62 (1.82)	1.54 (2.09)	2.30 (1.89)
	Nintendo 2^A, B^	18	1.28 (1.97)	.75 (1.71)	1.57 (1.96)	2.10 (1.55)
	Nintendo 3^B^	32	1.12 (1.83)	.86 (1.54)	1.43 (2.02)	2.05 (2.06)
	X-box^A^	34	1.93 (1.93)	.75 (1.70)	1.31 (2.27)	1.50 (2.06)
*Vehicles*			.80 (.99)	.81 (.82)	.41 (1.08)	.95 (1.01)
	School Bus*^C^*	1	−.61 (1.62)	−.22 (1.56)	−.77 (1.94)	−.35 (2.10)
	Sports Car 1	21	2.15 (1.94)	1.32 (1.59)	.77 (2.06)	1.95 (1.47)
	Sedan	23	.84 (1.61)	1.07 (1.37)	.89 (1.47)	.50 (1.54)
	SUV C	30	.52 (1.66)	.82 (1.33)	.43 (1.42)	1.50 (1.32)
	Sports Car 2	39	1.10 (1.71)	1.05 (1.42)	.71 (1.69)	1.15 (1.50)
*Construction^A, B^*		.22 (1.08)	−.40 (1.05)	.16 (1.11)	.20 (.84)	
	Bulldozer 1^A, B^	2	.25 (1.72)	−.88 (1.42)	−.17 (1.56)	.40 (.99)
	Bulldozer 2^A, B^	7	.24 (1.42)	−.53 (1.36)	.14 (1.88)	.35 (1.31)
	Tractor 1^A, B^	25	.16 (1.50)	−.27 (1.29)	.34 (1.26)	.35 (1.04)
	Forklift A	28	.16 (1.29)	−.43 (1.33)	.20 (1.28)	−.25 (1.16)
	Tractor 2	38	.28 (1.69)	.09 (1.32)	.29 (1.15)	.15 (1.73)
*Airplanes^A, B^*			1.03 (1.27)	−.13 (.99)	.63 (1.45)	1.03 (1.48)
	Plane 1^A, B^	9	.69 (1.60)	−.38 (1.42)	.34 (2.13)	1.30 (1.38)
	Jet^A, B^	19	1.04 (2.36)	−1.01 (1.67)	.97 (1.99)	.75 (2.17)
	Plane 2	20	.88 (1.75)	.25 (1.52)	.09 (2.01)	1.60 (1.73)
	Helicopter^A^	27	.99 (1.69)	−.10 (1.42)	.34 (1.75)	.80 (1.99)
	Shuttle^A, B^	29	1.57 (1.69)	.60 (1.45)	1.43 (1.79)	1.25 (2.15)
*Blocks*			.66 (1.17)	.86 (1.00)	.83 (1.10)	1.23 (1.22)
	Blocks 1	4	.82 (1.79)	1.07 (1.50)	1.14 (1.88)	1.35 (1.69)
	Legos	6	1.16 (1.66)	1.24 (1.30)	1.40 (1.26)	1.60 (1.60)
	Blocks 2	13	.52 (1.93)	.96 (1.62)	.86 (1.80)	1.45 (1.50)
	Blocks 3^C^	16	.57 (1.53)	.92 (1.29)	.43 (1.52)	1.15 (1.27)
	Blocks 4	22	.24 (1.29)	.13 (1.29)	.34 (1.33)	.60 (1.47)
*Clocks*			.46 (.99)	.17 (.79)	.31 (1.28)	.58 (1.19)
	Watch 1	5	−.01 (1.55)	−.53 (1.39)	−.57 (1.61)	.00 (1.69)
	Watch 2^A^	8	1.10 (1.66)	.34 (1.29)	.51 (1.82)	.85 (1.84)
	Big Ben	11	1.31 (1.77)	1.38 (1.48)	.97 (1.81)	.85 (1.79)
	Alarm Clock^B^	12	−.73 (1.66)	−.90 (1.66)	−.03 (1.90)	.30 (1.26)
	Watch 3	17	.61 (1.79)	.58 (1.27)	.69 (2.17)	.90 (1.83)
*Signs^A, B^*			−.38 (.98)	−.63 (.70)	−.29 (.74)	−.14 (.60)
	Interstate	3	.75 (1.71)	.50 (1.24)	.71 (1.30)	.25 (1.68)
	No U-Turn^B^	24	−.91 (1.41)	−1.16 (1.22)	−.57 (1.22)	−.35 (1.14)
	Dead End^A^	26	−1.13 (1.63)	−1.64 (1.41)	−1.06 (1.30)	−1.20 (1.44)
	Yield B	36	−.45 (1.35)	−.36 (1.12)	−.26 (1.20)	.40 (.88)
	One Way	40	−.16 (1.37)	−.48 (1.13)	−.29 (1.20)	.20 (1.06)

p<.05: A: Males>Females; B: ASD>TD; C: Females>Males; D: TD>ASD.

**Table 6:** Means and standard deviations for valence ratings on LAI images.

		Image #	TD Males	TD Females	ASD Males	ASD Females
*Clothing*			−.28 (.99)	−.22 (.75)	−.11 (.77)	.08 (.47)
	Pants^D^	2	−.51 (1.42)	−.55 (1.33)	−.28 (1.26)	.10 (1.07)
	Shirt	12	−.07 (1.35)	−.12 (1.07)	−.28 (1.34)	.05 (.94)
	Jeans	17	−.18 (1.64)	.17 (1.45)	−.06 (1.47)	.10 (1.48)
	Shoe	33	−.96 (1.74)	−.63 (1.60)	−.39 (1.08)	−.30 (1.26)
	Socks	34	.30 (1.61)	.04 (1.14)	.44 (1.21)	.45 (1.43)
*Outerwear*			.07 (.92)	.17 (.70)	.05 (.77)	.10 (1.14)
	Gloves	11	−.42 (1.83)	−.64 (1.33)	−.64 (1.33)	−.65 (1.73)
	Hat	15	.04 (1.77)	−.18 (1.17)	.06 (1.12)	.05 (1.47)
	Jacket^B^	16	.16 (1.44)	.74 (1.38)	.25 (1.44)	.25 (1.52)
	Sunglasses^B^	37	.51 (1.45)	.75 (1.22)	.08 (1.70)	.75 (1.29)
*Office Supplies* ^D^			.15 (.87)	.27 (.64)	.49 (.69)	.43 (.60)
	Pencils^B^	5	.70 (1.57)	1.77 (1.38)	.97 (1.48)	1.45 (1.79)
	Key	18	.55 (1.26)	.38 (1.12)	.25 (1.16)	.60 (.99)
	Lock^A, D^	21	−.39 (1.39)	−.49 (1.10)	.44 (1.32)	−.10 (1.33)
	Pens	26	.18 (1.45)	.18 (1.37)	.58 (1.54)	.25 (1.33)
	Scissors^A, D^	32	−.31 (1.10)	−.49 (1.23)	.22 (.99)	−.05 (.69)
*Kitchen Supplies* ^B, C^		.63 (1.06)	.86 (.98)	.44 (.95)	.50 (.87)	
	Bowl	30	1.19 (1.55)	1.18 (1.44)	.47 (1.30)	1.00 (1.45)
	Sponge	35	.09 (1.53)	.27 (1.29)	.31 (1.14)	−.25 (1.02)
	Teapot^B^	39	.61 (1.56)	1.12 (1.44)	.56 (1.32)	.75 (1.29)
*Furniture*			.47 (1.19)	.56 (.93)	.52 (.83)	.39 (.84)
	Chair	3	.91 (1.60)	.97 (1.33)	.81 (1.47)	.40 (1.31)
	Chest	4	.25 (1.56)	.20 (1.25)	.33 (.99)	.25 (1.41)
	Drawers	7	.27 (1.41)	.52 (1.08)	.36 (1.27)	−.05 (1.31)
	Table	38	.43 (1.44)	.55 (1.19)	.58 (1.20)	.95 (1.05)
*Tools* ^A, D^			.00 (1.00)	−.20 (.80)	.23 (.67)	.12 (.67)
	Flashlight^A^	9	.15 (1.10)	−.01 (.99)	.67 (1.59)	−.10 (1.07)
	Hammer^A, D^	14	.21 (1.43)	−.45 (1.41)	.33 (1.17)	.05 (1.47)
	Toolbox	22	−.16 (1.65)	−.29 (1.30)	.19 (1.14)	−.10 (1.29)
	Brush^B^	25	−.31 (1.46)	.25 (1.36)	−.03 (1.28)	.45 (1.79)
	Wrench^A, D^	40	.10 (1.50)	−.50 (1.20)	−.03 (.70)	.30 (1.66)
*Instruments* ^B^			.99 (1.22)	1.34 (1.08)	.81 (1.05)	1.39 (1.17)
	Drums	8	1.06 (1.44)	1.22 (1.31)	.58 (1.89)	1.30 (1.49)
	Guitar	13	1.30 (1.71)	1.55 (1.28)	1.06 (1.31)	1.60 (1.50)
	Piano	19	.73 (1.76)	1.01 (1.64)	.97 (1.59)	1.60 (1.70)
	Maracas^B, C^	23	.85 (1.73)	1.55 (1.43)	.64 (1.46)	1.05 (1.47)
*Plants* ^B, C^			.69 (1.25)	1.29 (1.01)	.44 (1.10)	.76 (.99)
	Flowers^B, C^	10	.93 (1.39)	1.74 (1.42)	.56 (1.46)	1.50 (1.76)
	Leaf^B, C^	20	1.09 (1.71)	1.97 (1.44)	.72 (1.63)	1.20 (1.40)
	Pine cone	27	.45 (1.61)	.51 (1.41)	.25 (1.44)	.10 (1.25)
	House Plant^B, C^	29	.30 (1.79)	.92 (1.50)	.25 (1.23)	.25 (1.33)

p<.05: A: Males>Females; B: Females>Males; C: TD>ASD; D: ASD>TD.

**Table 7:** Means and standard deviations for valence ratings on Social images.

		Image #	TD Males	TD Females	ASD Males	ASD Females
*Children* ^A, B^			.88 (1.49)	1.79 (1.09)	.48 (1.43)	1.08 (1.22)
	Infant 1^A, B^	1	1.75 (1.98)	2.45 (1.69)	.42 (2.21)	1.45 (1.82)
	Infant 2^A, B^	2	1.15 (1.73)	2.05 (1.55)	.64 (1.84)	1.55 (1.79)
	Infant 3^A, B^	3	1.03 (2.15)	2.13 (1.77)	.58 (1.93)	1.30 (1.75)
	Boy 1^A, B^	4	−.13 (1.73)	.71 (1.75)	−.28 (1.49)	.10 (1.68)
	Boy 2^A, B^	5	.75 (1.85)	1.81 (1.55)	.36 (1.48)	1.25 (1.62)
	Girl 1^A^	8	.69 (2.02)	1.75 (1.53)	.67 (1.72)	1.55 (1.61)
	Girl 2^A, B^	9	1.09 (2.04)	2.08 (1.45)	.81 (1.83)	1.20 (1.36)
	Girl 3^A, B^	10	.66 (1.74)	1.54 (1.50)	.61 (1.84)	.70 (1.69)
	Girl 4^A, B^	11	.91 (1.64)	1.60 (1.20)	.56 (1.32)	.60 (1.31)
*Adult Men* ^A^			−.14 (.95)	.14 (.80)	−.24 (1.21)	−.81 (.62)
	Man 1^B^	6	1.01 (1.78)	.91 (1.65)	−.11 (1.94)	.05 (1.10)
	Man 2	7	.70 (1.70)	.63 (1.38)	.22 (1.88)	.70 (1.34)
	Man 3	12	−.27 (1.52)	−.25 (1.29)	−.36 (1.55)	.05 (1.00)
	Man 4	13	.01 (1.81)	.24 (1.33)	−.22 (1.46)	.20 (1.28)
	Man 5	14	−.45 (1.40)	−.34 (1.22)	−.36 (1.33)	.15 (1.04)
	Man 6	15	−.81 (1.70)	−.79 (1.38)	−.53 (1.56)	−.30 (1.45)
	Man 7^A^	16	−1.24 (1.57)	−.67 (1.65)	−.72 (1.80)	−.25 (1.58)
	Man 8	17	−.25 (1.58)	.00 (1.35)	−.22 (1.51)	.10 (1.17)
	Man 9^A^	18	−.25 (1.87)	.45 (1.60)	−.28 (1.70)	−.05 (1.32)
	Man 10^A^	19	−.19 (1.79)	.42 (1.42)	−.17 (1.58)	−.05 (.83)
	Man 11^B^	20	.42 (1.87)	.53 (1.44)	.11 (1.82)	−.60 (1.70)
	Man 12^A, B^	21	.15 (1.40)	.93 (1.42)	−.11 (1.45)	.05 (.89)
	Man 13	22	−.24 (1.62)	−.06 (1.43)	−.44 (1.66)	−.30 (.80)
	Man 14	23	−.34 (1.56)	−.25 (1.23)	−.11 (1.39)	−.45 (.89)
	Man 15^B^	24	.43 (1.43)	.71 (1.39)	.08 (1.38)	−.55 (1.54)
	Man 16^A^	25	−.93 (1.81)	−.27 (1.74)	−.69 (1.45)	−.05 (1.67)
*Adult Women* ^B^			.62 (1.07)	.57 (.78)	.21 (1.06)	.26 (.73)
	Woman 1^A, B^	26	.58 (1.37)	1.12 (1.14)	.25 (1.18)	.10 (1.37)
	Woman 2^A^	27	−.10 (1.85)	.36 (1.17)	.00 (1.31)	−.05 (1.10)
	Woman 3	28	.52 (1.46)	.42 (1.10)	.33 (1.55)	−.05 (1.57)
	Woman 4^A, B^	29	.93 (1.41)	1.30 (1.35)	.36 (1.36)	.85 (1.98)
	Woman 5^B^	30	.67 (1.32)	.72 (1.13)	.08 (1.34)	.70 (.92)
	Woman 6	31	.64 (1.46)	.60 (1.14)	.33 (1.12)	.70 (1.45)
	Woman 7^B^	32	1.84 (1.61)	1.10 (1.16)	.64 (1.61)	1.30 (1.75)
	Woman 8	33	.34 (1.60)	.59 (1.11)	.28 (1.06)	.35 (.88)
	Woman 9	34	−.30 (1.70)	−.02 (1.28)	−.35 (1.36)	.50 (1.40)
	Woman 10^B, C^	35	1.24 (2.03)	.50 (1.72)	.42 (1.83)	−.25 (1.97)
	Woman 11^B,^ ^C^	36	1.73 (1.67)	.65 (1.24)	.33 (1.51)	.05 (1.96)
	Woman 12	37	.21 (1.34)	.29 (1.31)	−.03 (1.36)	.25 (.91)
	Woman 13	38	.04 (1.59)	.34 (1.49)	−.28 (1.51)	−.30 (1.38)
	Woman 14^C^	39	.57 (2.130	−.19 (1.44)	.25 (1.56)	−.50 (1.36)
	Woman 15	40	.45 (1.55)	.79 (1.24)	.36 (.90)	.25 (.72)

p<.05: A: Females>Males; B: TD>ASD; C: Males>Females.

**Figure 2:**
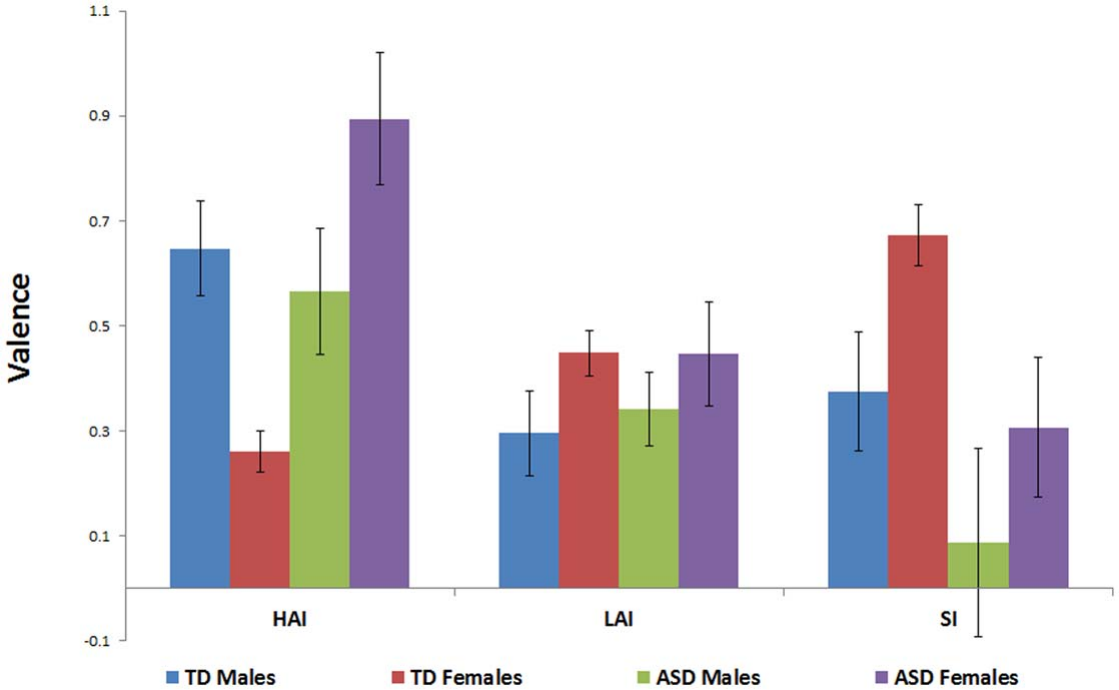
Valence ratings for social images, HAI objects and LAI objects, by group and gender.

**Figure 3:**
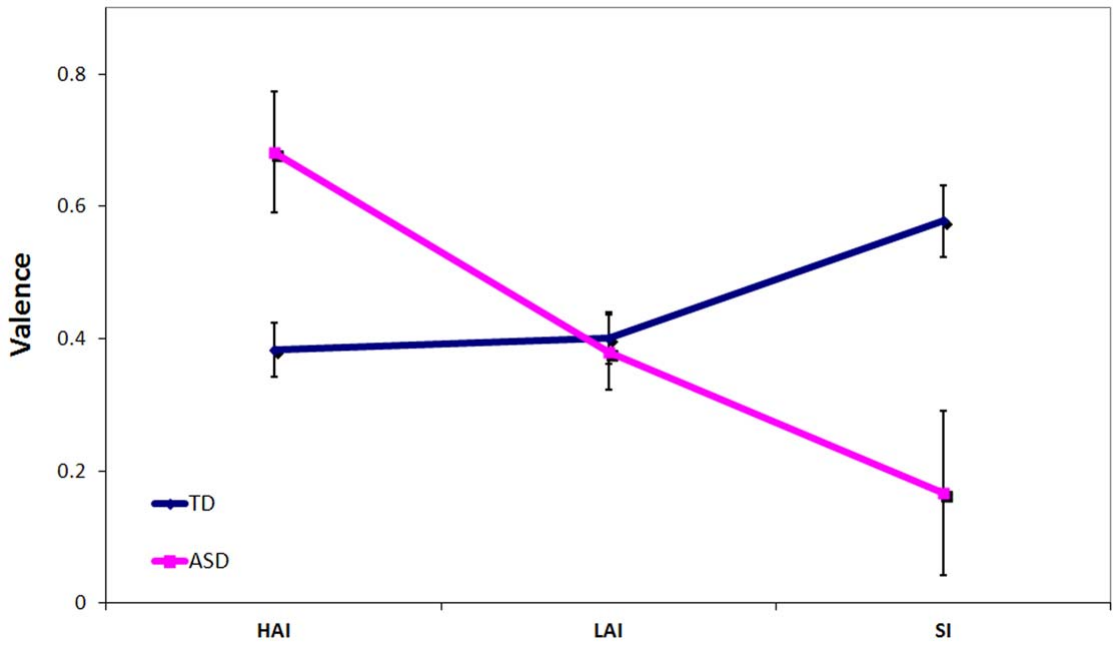
Significant group x image type interaction for valence ratings.

A main effect of image type was found (F (1.69, 447.08) = 10.31, p<.001, eta squared = .04; see Figure 2). Post hoc paired-samples t-tests revealed that social images were rated higher overall on valence than LAI images (t (269) = 1.34, p = .04), but ratings for HAI images did not differ significantly from either LAI images (p = .18) or social images (p = .40). No main effects of group (p = .91) or sex (p = .15) were found, nor was the interaction between group and sex significant (p = .23). However, a significant interaction emerged between group and image type (F (1.69, 447.08) = 14.37, p<.001, eta squared = .05; see Figure 3) that was driven by the ASD group providing higher valence ratings for HAI images (t (267) = 3.23, p = .01) and lower ratings for social images (t (267) = 3.33, p = .001) than the TD group, while the two groups did not differ on ratings of LAI objects (p = .78). The sex by image type interaction was significant (p = .048), with males providing higher valence ratings than females for HAI objects (t (167.66) = 3.38, p = .001), but lower valence ratings for LAI objects (t (267) = 2.01, p = .046) and social images (t (267) = 3.42, p = .001). A three-way interaction between group, sex and image type also emerged (F (1.69, 447.08) = 7.95, p = .001, eta squared = .03; see Figure 2) that was driven by different group x sex patterns for each image type: for HAI images, ASD females provided higher ratings than did TD females (t (164) = 5.44, p<.001) and trended towards providing higher ratings than ASD males (t (54) = 1.76, p = .08), while TD males provided higher ratings than did TD females (t (91.86) = 3.89, p<.001) but not ASD males (p = .59); for LAI images, a trend emerged for TD females to provide higher ratings than TD males (p = .07) but not ASD females (p = .99), while ASD males and TD males did not differ from each other (p = .71), nor did ASD males from ASD females (p = .38); for social images, TD females provided higher ratings than TD males (t (211) = 2.57, p = .01) and ASD females (t (164) = 2.20, p = .03), while ASD males and TD males did not differ from each other (p = .16), nor did ASD males from ASD females (p = .41).

*Discrepancy Ratings & Correlation with Clinical Features:* A multivariate ANOVA with sex as a covariate revealed that the ASD and TD groups differed in discrepancy ratings between social and HAI images for valence (F (1, 266) = 17.29, p<.001, eta squared = .06) but not arousal (F (1, 266) = .002, p = .97, eta squared<.001). Within the TD group, AQ was negatively associated with discrepancy scores for valence (i.e., the higher the AQ score, the lower the valence preference for social relative to HAI images; r = −.18, p = .008) but not for arousal (r = −.01, p = .91). Within the ASD group, AQ was also negatively correlated with valence discrepancy scores (r = −.42, p = .001), but not correlated with arousal discrepancy scores (r = .18, p = .18).

## Discussion

Although circumscribed interests (CI) are a nearly ubiquitous characteristic of ASD [1] [6], there is little empirical research addressing this symptom domain. The current study found that adults with ASD differed from TD comparison participants in their affective responses to images related to CI compared to both social images and non-CI images. Subjective emotional experiences of three novel image sets were assessed using ratings of valence and arousal derived from circumplex models of emotion described by a number of theorists to reflect activity of two distinct and fundamental neural systems critical for responding to behaviorally relevant environmental stimuli [47] [61] [62].

While the ASD and TD groups did not differ in arousal ratings of CI-related images, non-CI related images or social images, significant differences emerged for valence ratings. Individuals with ASD rated CI-related images as more positively experienced, and social images as less positively experienced, than TD individuals. These results suggest that HAI images, and by extension, CI in ASD reflect higher subjective ratings of pleasure that likely contributes to their increased salience, and may help explain previous research indicating that CI-related images disproportionately capture attention [18] [19] and hyperactivate neural circuitry subserving reward processing [16] in ASD. Collectively, these studies span a broad range of methods, from perceptual to neural to self-reported affective experiences, and present converging evidence of abnormal cognitive-affective reward mechanisms underlying CI in ASD.

If present from early in life, such processes could have important developmental repercussions. A reward system that is biased away from social information in favor of nonsocial aspects of the environment may result in reduced social motivation and increased interest and restricted activity with circumscribed nonsocial experiences. As social proficiencies are developmentally constructed through transactional brain-behavior interactions [26], this model of abnormal cognitive-affective reward might help explain the emergence of social deficit in ASD, as well as the presence of certain aspects of repetitive behavior. The current study supports this model by reporting elevated positivity towards CI-related objects in ASD but decreased positivity towards social stimuli. Thus, affective responses in ASD are not abnormal in a domain-general sense, but rather reflect a bifurcated pattern of response to social and specific nonsocial content.

We also observed stark sex differences in valence ratings of HAI images. TD males gave higher valence ratings of HAI images than TD females, suggesting that these types of objects are associated with male-typical preferences. Indeed, although TD males provided higher valence ratings on social images relative to males with ASD, their valence ratings on HAI images were similar. This is consistent with the developmental literature indicating that from a very young age, males exhibit greater interest in mechanically-related toys and objects [56] [63], qualities that disproportionately manifest in extreme interests in TD boys [57] and dominate the content of CI in ASD [7]. This sex difference in object preferences is hypothesized to be related to prenatal androgen exposure [64]. For example, variability in prenatal androgen exposure is associated with male-typical play and behavior in childhood [65], and studies of females with congenital adrenal hyperplasia who are exposed prenatally to abnormally high amounts of androgens demonstrate greater interest in masculine toys and less interest in feminine toys relative to female controls, independent of any socialization differences [66] [67].

Females with ASD in the current study differed markedly in their valence preferences for CI-related objects compared to TD females. The more male-typical profile they present coheres with previous findings of lack of female-typical play preferences in ASD girls [68] and the presence of increased prevalence of masculine characteristics and conditions [69]. Future work investigating sex differences in CI profiles in ASD is therefore encouraged. Finally, it is worthy of note that females with ASD also provided lower arousal ratings compared to males with ASD, as well as TD females and males. This finding was not anticipated and thus may warrant further investigation.

Even after co-varying for sex differences, however, the ASD group differed from the TD group in this study by exhibiting higher discrepancy preferences for HAI images compared to social images. A relative bias for selectively favoring certain nonsocial over social content, as evidenced by the valence discrepancy data reported here, is indicative of preference and thus may be more clinically meaningful than absolute differences on a single stimulus type. For instance, greater affective responses evoked by certain nonsocial aspects of the environment relative to social stimuli may result in the experiential prioritization of a restricted range of environmental input. This process may contribute to non-normative experiences that lead to deleterious consequences for neural and social development [26]. Further, because higher relative valence ratings for HAI images to social stimuli was also associated with the presence of autistic traits within the TD population, an affective bias to certain nonsocial stimuli relative to social stimuli may relate broadly to autism-related characteristics, existing even at subclinical levels of autistic symptomotology.

This study is not without limitations. First, the sample was limited to adult participants. Because the HAI images presented in this study were selected based upon profiles of common CI in children [9] and their visual attentional preferences [19], the included images may not have reflected the most representative content of CI for adults. For instance, HAI categories used in this study that may retain interest into adulthood (e.g., trains) differentiated ASD from TD in valence ratings far better than HAI categories that may be more specific to CI during childhood (e.g., blocks). Future research that obtains affective ratings on these images in ASD children might result in even greater effects. Similarly, the selection of images that target CI content more age-appropriate for ASD adults may also elicit larger discrimination between groups, although the inclusion of such images is difficult because, at the present time, the content of CI in adults with ASD has not been empirically investigated as has been done with children. Further, the effects reported in this study occur only at the level of group differences. Individuals with ASD may exhibit a restricted range of interest to only a subset of the CI categories included here, or even to none at all. Studies examining individual differences in the affective component of CI in ASD, perhaps by utilizing exemplars of individual-specific CI, could prove valuable.

Second, we did not have access to the full clinical records of the ASD sample. While all included participants held or reported clinical diagnoses of ASD and exhibited significant autism symptoms as measured by the AQ, follow-up studies should include ADOS and ADI characterization and ask about the presence of non-ASD psychopathology. Further, because the sample consisted of individuals who were capable of completing an online survey and self-reported to be high functioning, we do not yet know whether our findings would extend to lower-functioning individuals. The fact that CI are similarly prevalent in high and low functioning individuals with ASD [3] suggests that generalized results might be anticipated, but perhaps with qualitative distinctions. For example, it is unknown whether the content of the HAI images included here reflect common targets of CI in low functioning individuals, as they were selected based upon eye-tracking profiles from high functioning participants. Future work may also seek to extend beyond including only TD individuals as a comparison group. Because CI are a highly prevalent characteristic of ASD that may represent a discriminating characteristic of the disorder, studies that include a comparison group with another neuropsychiatric disorder, especially those characterized by repetitive and restricted behaviors (e.g., Obsessive-Compulsive Disorder), may provide further evidence that elevated affective responses to CI-related images is a characteristic specific to ASD and not reflective of general developmental abnormality. Finally, only static images were used in this study. Given that CIs in ASD commonly involve mechanical parts [7], future research that employs more ecologically valid exemplars of CI, particularly those that highlight their dynamic elements, might be expected to elicit larger group differences in affective responses. Whether dynamic properties would differentially affect valence and arousal judgments or influence ratings of social stimuli remain open questions.

Despite these limitations, the present study furthers our understanding of CI by providing evidence that they elicit a heightened pleasure response in ASD. The group differences reported here suggest that the differing visual attention and reward circuitry responses to CI and social images previously found in ASD [16] [17] [18] may be related to a distinct pattern of affective responses. Thus, we are providing evidence of a critical link between endophenotypic measures and clinical presentation by highlighting atypical subjective emotional experiences in ASD that may contribute to the elevated reward circuitry responses to CI, and to reduced reward circuitry responses to social stimuli. Additionally, the current study examines sex differences, an understudied aspect of ASD research, and also reports evidence that autism-related characteristics predict higher valence ratings for CI relative to social images, both within ASD and TD populations. Finally, this paper describes the creation and validation of a normative collection of pictorial stimuli that is designed specifically to facilitate research aimed at understanding subjective, behavioral, and neurobiological correlates of CI in autism. While the original intent of the stimulus set was to aid investigations of CI and attention in ASD, the images may have broader use as they not only elicit discrepant responses in ASD and TD populations but also evoke large sex differences. The image set is available to the scientific community at http://can.unc.edu/content/site/resources/ and we hope that other investigators will select specific images that relate to their own research interests based on normative valence and arousal ratings presented here.
